# Advancing Neuropharmacology and Neurodegenerative Disease Therapy: Bridging Gaps and Paving New Pathways

**DOI:** 10.3390/ph18050606

**Published:** 2025-04-22

**Authors:** Manuela Leri, Marzia Vasarri

**Affiliations:** Department of Experimental and Clinical Biomedical Sciences, University of Florence, Viale Morgagni 50, 50134 Florence, Italy

The Special Issue of *Pharmaceuticals*, titled “Multi-target drug treatments for neurodegenerative disease”, highlighted recent advancements in neuropharmacology and the therapeutic landscape for neurodegenerative diseases, representing a significant stride forward in our understanding of these complex conditions. As we synthesize the contributions of various studies, it is evident that despite our remarkable progress, notable knowledge gaps persist. This Editorial aims to summarize these insights and outline future research trajectories for subsequent investigations in addressing neurological disorders.

Neurodegenerative diseases, notably Alzheimer’s disease (AD) and neurodegeneration with brain iron accumulation (NBIA), are characterized by intricate molecular pathways that contribute to their pathophysiology. For instance, Iqbal et al. have underscored the potential of a multi-targeted approach to inhibit GSK3β, NMDA-receptor, and BACE-1, which are crucial in the development of AD [1]. Their findings reveal the interplay between β-secretase enzyme activity and amyloid plaque deposition, linking oxidative stress to Tau phosphorylation and neurofibrillary tangles. This understanding is pivotal for the development of combinatory therapeutic strategies, enabling us to address multiple mechanisms simultaneously [1].

Additionally, Angarita-Rodríguez et al. have opened new avenues by investigating enzymatic metabolic switches in astrocytes as potential therapeutic targets for neurological disorders [2]. Their analysis integrates protein interaction networks and in silico modeling, emphasizing the polypharmacological potential of existing drugs, setting the stage for future drug repurposing studies. Such insights underscore a paradigm shift emphasizing cellular models and metabolic pathways rather than a solely receptor-focused approach.

The research by Álvarez-Córdoba et al. extends this discussion by advocating for the use of patient-derived cellular models in precision medicine, particularly for conditions like pantothenate kinase-associated neurodegeneration, which currently lacks effective treatments. Their findings highlight the urgent need for personalized therapies based on specific genetic profiles, underscoring the need for a deeper understanding of the specific mechanisms driving neurodegenerative diseases [3].

Moreover, Stanciu et al. have explored the potential of combining sodium/glucose cotransporter 2 (SGLT2) inhibitors, such as Canagliflozin, with Donepezil for AD treatment. Their findings suggest that this therapeutic strategy may enhance neuroprotection through a variety of mechanisms, including brain-derived neurotrophic factor (BDNF) modulation and amyloid burden reduction. The promising outcomes from their in vivo murine studies pave the way for clinical research, reinforcing the multifactorial nature of effective AD management through combination therapies [4].

Furthermore, the COVID-19 pandemic has exacerbated incidences of dementia and AD, prompting urgent explorations into the interplay between viral infections and neurodegenerative diseases. Navolokin et al. emphasize the need to investigate strategies to mitigate amyloid deposition associated with COVID-19, marking a critical intersection of two major health crises [5]. The alarming increase in AD-related deaths during the pandemic highlights the need for neuroprotective strategies to be prioritized within public health frameworks.

While these contributions provide a robust understanding of the landscape, significant knowledge gaps remain, particularly in the translation of preclinical findings to clinical practice. Future research should focus on several key areas: elucidating the mechanistic regulation of neural pathways in degenerative diseases, employing patient-specific models in preclinical studies, and conducting rigorous clinical trials to evaluate the efficacy of combination therapies.

In conclusion, this Special Issue presents a compendium of significant research that not only enriches our understanding of the molecular and cellular mechanisms underlying neurodegenerative diseases but also highlights avenues that require further exploration. As we move forward, continued innovation and interdisciplinary collaboration will be essential in unraveling the complexities of these conditions and improving therapeutic outcomes for affected individuals. The road ahead is filled with promise, and it is our collective responsibility to harness these insights to forge a brighter future for individuals affected by neurological disorders.
